# The application of HPLC and microprobe NMR spectroscopy in the identification of metabolites in complex biological matrices

**DOI:** 10.1007/s00216-015-8556-y

**Published:** 2015-03-27

**Authors:** Zhaoxia Miao, Mengxia Jin, Xia Liu, Wei Guo, Xiangju Jin, Hongyue Liu, Yinghong Wang

**Affiliations:** State Key Laboratory of Bioactive Substances and Functions of Natural Medicines, Institute of Materia Medica, Chinese Academy of Medical Sciences & Peking Union Medical College, Beijing, 100050 China

**Keywords:** High-performance liquid chromatography, NMR, Urine, Feces, Metabolite identification

## Abstract

Nuclear magnetic resonance (NMR)-based metabolomics can be used directly to identify a variety of metabolites in biological fluids and tissues. Metabolite analysis is an important part of life science and metabolomics research. However, the identification of some metabolites using NMR spectroscopy remains a big challenge owing to low abundance or signal overlap. It is important to develop a method to measure these compounds accurately. Two-dimensional NMR spectroscopy, metabolite prediction software packages, and spike-in experiments with authentic standards are often used to solve these problems, but they are costly and time-consuming. In this study, methods were developed to identify metabolites in complex biological mixtures using both high-performance liquid chromatography (HPLC) and off-line microprobe NMR spectroscopy. With use of these methods, 83 and 73 metabolites were identified in Sprague Dawley rat urine and feces, respectively. Among them, 40 and 45 metabolites, respectively, could not be identified with traditional NMR methods. Our research revealed that the combination of HPLC and NMR techniques could significantly improve the accuracy of trace and overlapped metabolite identification, while offering an effective and convenient approach to identify potential biomarkers in complex biological systems.

**Electronic supplementary material:**

The online version of this article (doi:10.1007/s00216-015-8556-y) contains supplementary material, which is available to authorized users.

## Introduction

Metabolomics is used to determine the metabolic profile of biological samples, identify specific biomarkers, and explore possible metabolic pathways. It has been used during drug development [1], and in clinical disease research [2, 3], pathology [4], toxicology [5] and nutrition studies [6]. Metabolomics mainly utilizes NMR spectroscopy [7], liquid chromatography (LC)–mass spectrometry [8] and gas chromatography–mass spectrometry [9] to analyze and evaluate biological specimens. Each analytical technique has its own advantages and shortcomings; none of them can be used individually to systematically and accurately identify metabolites in complex biological matrices. Since accurate metabolite identification directly determines the usefulness of the metabolomic analysis, metabolite identification has gained increased attention from the metabolomics research community. ^1^H NMR spectroscopy is often used for metabolomics research. As all ^1^H nucleuses have the same sensitivity, the reproducibility of NMR spectroscopy is typically high. In addition, specimens do not go through complex processing, and can be measured in the physiological state. Nevertheless, the limited spectroscopic dispersion of ^1^H NMR, about 12 ppm, results in a high degree of overlap of metabolite signals. Moreover, the signals of trace-level metabolites are often too weak and unclear to be accurately assigned, even if those signals do not overlap with other signals. Since some metabolites with low concentrations are indicative of certain disease states [10], it is important to measure these metabolites with certainty.

In NMR-based metabolomics research, metabolite identification is done mainly through the combined statistical correlation between the experimental results and those reported in the literature [11, 12], in databases (Madison Metabolomics Consortium Database, Human Metabolome Database, etc.) [13, 14], by metabolite prediction software packages (e.g., Chenomx NMR Suite) [15, 16], and by related two-dimensional NMR spectroscopy [17–21]. Two-dimensional NMR spectroscopy is arguably the most important spectroscopic technique for the elucidation of structures [22]; however, even if two-dimensional NMR spectroscopy is used, assignments are often challenging since signal overlap is extensive. The method of multiple spike-in of authentic standards is also used in metabolomics research [16]; however, standards may be expensive and difficult to obtain.

High-performance LC (HPLC) can be used to reduce the complexity of NMR spectra, and to increase the signal strength of trace-level compounds. Preliminary HPLC enrichment or purification, although time-consuming, is sometimes necessary to establish the structural identities of metabolites with low concentrations. For example, Liu et al. [23] identified three polyphenolic compounds from a well-researched plant, *Origanum vulgare* L, using LC–diode-array detection–solid-phase extraction (SPE)–cryo-NMR spectroscopy/mass spectrometry techniques. The coupling of NMR spectroscopy and HPLC has been applied not only in the analysis of complex mixtures of natural products [24–26], but also in the study of biological matrices. For example, Rezzi et al. [27] developed a new method of combining HPLC with NMR spectroscopy, and applied it to separate and identify 72 metabolites in human urine, and to identify felinine in cat urine. Akira et al. [28] used an LC–NMR approach to isolate and identify a previously unknown compound, succinyltaurine, in hypertensive rat urine. Aranibar et al. [29] applied HPLC and NMR spectroscopy to elucidate the previously unknown metabolites 1-methylhistidine and 3-methylhistidine as potential biomarkers of drug-induced skeletal muscle toxicity and hypertrophy in rats. In this study, microprobe NMR spectroscopy combined with HPLC was applied to improve metabolite identifications in Sprague Dawley rat urine and feces. The use of a microprobe provides a more convenient way to measure and quantify biological samples with a limited volume/mass/cell count [30, 31]. Therefore, our method could significantly shorten the time for sample separation, enrichment, concentration, and collection, and reduce the number of animals required.

In urine, most endogenous metabolites are polar small molecules, having poor retention on reversed-phase columns, leading to co-elution of compounds corresponding to chromatographic peaks. Therefore, a hydrophilic interaction LC (HILIC) column was used for separation of polar compounds [32]. Sprague Dawley rats have been widely used for NMR-based metabolomics research [33–35]. The choice of their urine and feces as biological samples is justified by their potential for further application in NMR-based metabolomics.

## Experimental

### Chemicals and reagents

Sodium 3-(trimethylsilyl)propionate-2,2,3,3-*d*_4_ (batch no. 1668-E) was purchased from Merck. Deuterium oxide (D_2_O) was purchased from Cambridge Isotope Laboratories. Methanol and acetonitrile (HPLC grade) were purchased from Beijing Chemical Works. Water (HPLC grade) was purchased from Hangzhou Wahaha Group. Dipotassium hydrogen phosphate (K_2_HPO_4_·3H_2_O), sodium dihydrogen phosphate (NaH_2_PO_4_·2H_2_O), and hydrochloric acid (analytical grade) were purchased from Beijing Chemical Works.

### Instruments

The NMR instrument (AVANCE III-500), equipped with a 1.7-mm NMR microprobe, was from Bruker. The HPLC system, equipped with a device for fraction collection (LC-20A), was from Shimadzu. The high-speed centrifuge (Sartorius Sigma 1-14) was purchased from Sigma. The freeze dryer (FDU-1100) was from Tokyo Rikakikai. The nitrogen evaporator (UGC-36 M) was from Beijing Yousheng. The pH meter (MP511) was from Shanghai Sanxin. The analytical balance (BT 124S) was from Sartorius.

### Sample preparations

Male Sprague Dawley rats (each weighing about 200 g) were purchased from Vital River Laboratory Animal Technology, Beijing, China (license no. SCXK Beijing 2012-0001). All protocols in this study were in accordance with regulations for the care and use of animals in research implemented by the National Institutes of Health. During the whole acclimatization and study period, all rats had access to food and water ad libitum, and were maintained on a 12 h light/dark cycle (21 ± 2 °C with a relative humidity of 45 ± 10 %). After a 7-day acclimatization period, rats were placed in metabolic cages, and their urine and feces were collected for 24 h. Urine was centrifuged at 12,000 revolutions per minute for 10 min to remove solids, and the supernatant was collected. Urine supernatant (1 mL) was stored at -20 °C for NMR measurement, and the remaining material was freeze-dried to provide urine powder for further analysis. Feces was stored at -20 °C for further analysis.

K_2_HPO_4_·3H_2_O (3.4233 g) and NaH_2_PO_4_·2H_2_O (2.3402 g) were dissolved in 10 mL D_2_O to prepare 1.5 M phosphate-buffered saline. The pH was adjusted to 7.40, and 0.0500 g sodium 3-(trimethylsilyl)propionate-2,2,3,3-*d*_4_ was added as an internal standard. The resulting solution was diluted tenfold to provide 0.15 M phosphate-buffered saline.

Urine supernatant (180 μL) and 1.5 M phosphate-buffered saline (20 μL) were transferred into a 0.6-mL microcentrifuge tube and centrifuged at 12,000 revolutions per minute for 10 min. The supernatant (60 μL) was transferred into a 1.7-mm NMR tube for NMR measurement. Urine powder (0.8 g) was dissolved in 2 mL water of pH 3.0 (pH adjusted with hydrochloric acid) and centrifuged at 12,000 revolutions per minute for 10 min. The supernatant was filtered through a 0.45-μm membrane filter before it was used for HPLC analysis.

Feces (0.4 g) and 0.15 M phosphate-buffered saline (1 mL) were placed in a mortar and ground to a suspension. The suspension was transferred into a 1.5-mL microcentrifuge tube and centrifuged at 12,000 revolutions per minute for 10 min. The supernatant (60 μL) was transferred into a 1.7-mm NMR tube for NMR measurement. Feces (4 g) and water (10 mL) were placed in a mortar and ground to a suspension. The mixture was transferred into 1.5-mL microcentrifuge tubes, and centrifuged at 12,000 revolutions per minute for 10 min. The supernatant (2 mL) was filtered through a 0.45-μm membrane filter before HPLC analysis.

### HPLC analysis methods

The preconcentrated urine (as described in the previous section, 100 μL) was analyzed using a HILIC analytical column (Shimadzu, 5 μm, 4.6 mm × 250-mm inner diameter) equipped with a HILIC cartridge guard column (Shimadzu, 5 μm, 4.6 mm × 10-mm inner diameter). Separation was achieved in 50 min at 40 °C with a flow rate of 1 mL/min. The mobile phase consisted of water (solvent A) and acetonitrile (solvent B) and the elution gradient was 95 % solvent B for 20 min followed by 95 % solvent B to 65 % solvent B in 30 min. Fractions were collected at 1-min intervals, for a total of ten injections. The fractions were each evaporated under a stream of nitrogen to remove acetonitrile. The remaining aqueous residue from each fraction was lyophilized in a freeze dryer.

The aqueous feces extract (100 μL) was analyzed using HPLC with a C_18_ analytical column (5 μm, 4.6 mm × 250-mm inner diameter) equipped with a C_18_ cartridge guard column (5 μm, 4.6 mm × 10-mm inner diameter ), both purchased from Shanghai Puning Analytical Technology. Separation was achieved in 30 min at 30 °C with a flow rate of 1 mL/min. The mobile phase consisted of water (solvent A) and methanol (solvent B) with a gradient elution of 0 % solvent B for 10 min followed by 0 % solvent B to 90 % solvent B in 20 min. Fractions were collected at 1-min interval, for a total of ten injections. The fractions were each evaporated under a stream of nitrogen to remove methanol. The remaining aqueous residue from each fraction was lyophilized in a freeze dryer.

### NMR analysis

Each freeze-dried urine and feces fraction was dissolved in 70 μL 0.15 M phosphate-buffered saline and transferred into a 1.7-mm NMR tube for NMR measurement.

Nuclear Overhauser effect spectroscopy (NOESY) pulse sequence (recycle delay–90°–*t*_1_–90°–*t*_m_–90°–acquisition), ^1^H–^13^C heteronuclear single quantum correlation (HSQC) spectroscopy, and ^1^H–^1^H homonuclear total correlation spectroscopy (TOCSY) spectra were collected for each fraction at 25 °C. For the NOESY pulse sequence, a total of 128 transients were collected into 81,920 data points for each spectrum with a spectroscopic width of 16 ppm and a recycle delay of 4.0 s. The mixing time (*t*_m_) was 100 ms, and the acquisition time was 5.12 s. For HSQC spectroscopy, 128 increments with 256 transients per increment were collected into 1,024 data points with a spectroscopic width of 5,000 and 26,000 in the first and second dimensions, respectively. The coupling constant (*J*) was set at 145 Hz. The TOCSY NMR spectra were acquired with 128 transients per increment, with 256 increments collected into 2,048 data points, using the MLEVPHPP pulse program with a mixing time of 80 ms. A line-broadening factor of 0.3–1.0 Hz was applied to the free induction decay before Fourier transformation.

## Results

**Fig. 1:**
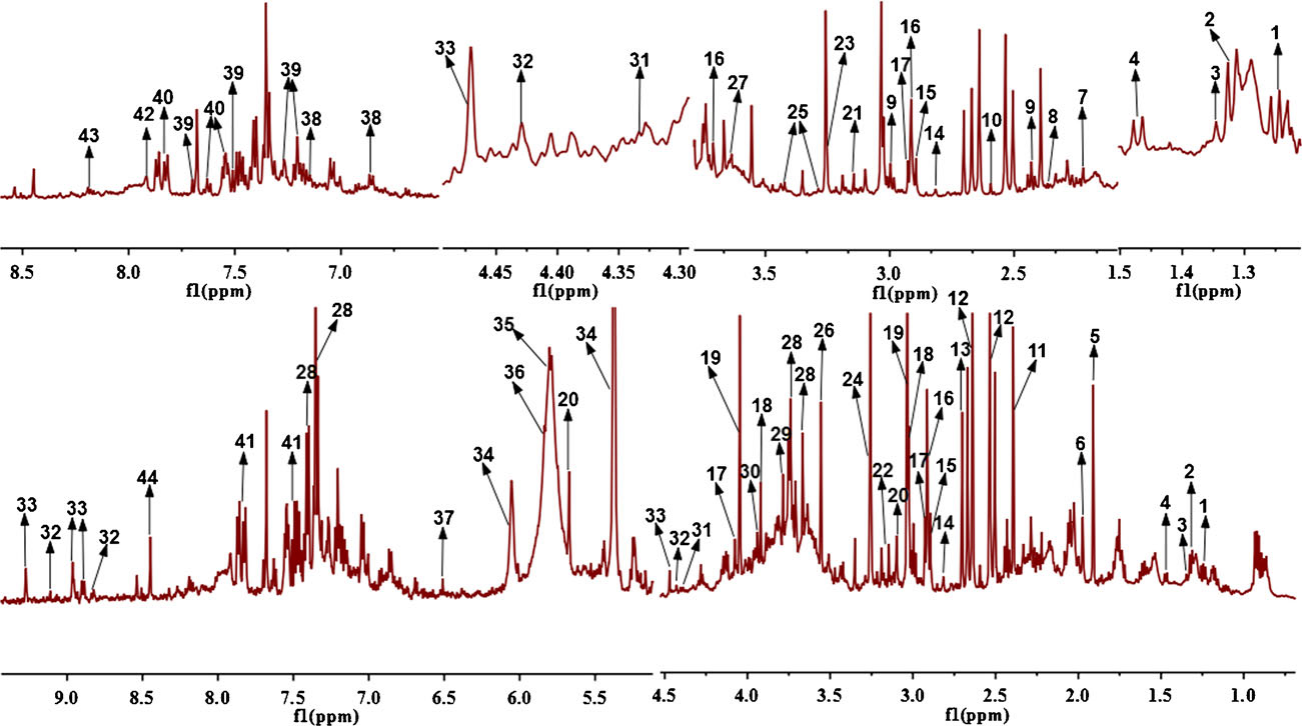
Typical ^1^H nuclear Overhauser effect spectroscopy (NOESY) spectra of rat urine. *1* 3-hydroxyisovalerate, *2* lactate; *3* 2-hydroxyisobutyrate, *4* alanine, *5* acetate, *6* acetamide, *7* acetone, *8* pyruvate, *9* 2-oxoglutarate, *10* methylamine, *11* succinate, *12* citrate, *13* dimethylamine, *14* methylguanidine, *15* trimethylamine, *16 N*,*N*-dimethylglycine, *17 N*-methylhydantoin, *18* creatine, *19* creatinine, *20 cis*-aconitate, *21* malonate, *22* choline, *23* betaine, *24* trimethylamine *N*-oxide, *25* taurine, *26* glycine, *27* ethylene glycol, *28 N*-phenylacetylglycine, *29* guanidoacetate, *30* glycolate, *31* tartrate, *32* trigonelline, *33* 1-methylnicotinamide, *34* allantoin, *35* uracil, *36* urea, *37* fumarate, 38 4-hydroxyphenylacetate, *39* 3-indoxylsulfate, *40* hippurate, *41* benzoate, *42* xanthine, *43* hypoxanthine, *44* formate

**Fig. 2:**
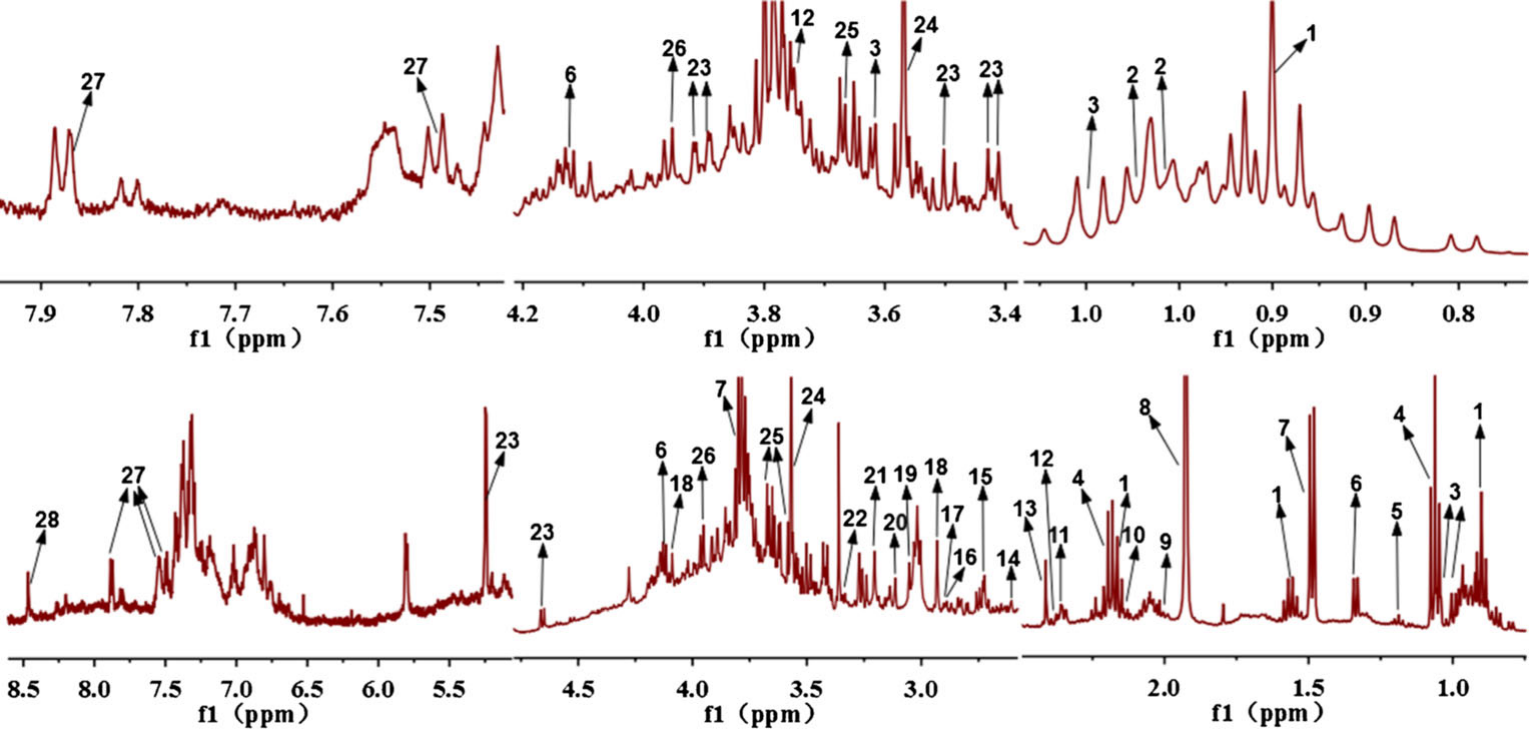
Typical ^1^H NOESY spectra of water-extracted rat feces. *1* butyrate, *2* leucine, *3* valine, *4* propionate, *5* ethanol, *6* lactate, *7* alanine, *8* acetate, *9* acetamide, *10* methionine, *11* glutamate, *12* pyruvate, *13* succinate, *14* methylamine, *15* dimethylamine, *16* methylguanidine, 17 trimethylamine, *18 N*-methylhydantoin, *19* creatinine, *20* malonate, *21* choline, *22* proline, *23* glucose, *24* glycine, *25* glycerol, *26* glycolate, *27* benzoate, *28* formate

The resonances were assigned to specific metabolites according to assignments reported in the literature, including the 500-MHz library from Chenomx NMR Suite 7.5 (Chenomx, Edmonton, AB, Canada) and the Human Metabolome Database. NMR analysis of the HPLC (HILIC and C_18_) fractions identified 83 and 73 metabolites in rat urine and feces, respectively (Tables 1, 2, Figs. S1–S8), whereas 40 and 45 metabolites, respectively, could not be identified in the NMR spectra of non fractionated urine and feces samples (called “the reference urine profile” and “the reference feces profile,” respectively; Figs. 1, 2).

**Table 1:** ^1^H NMR chemical shift assignment of metabolites in urine fractions

Metabolite	Assignment	*δ* ^1^H (ppm) and multiplicity
Acetate^a^	CH_3_^e^	1.92 (s)
Dimethylamine^a^	CH_3_^e^	2.73 (s)
3-Indoxylsulfate^b^	C_2_H^f^, C_4_H^f^, C_5_H^f^, C_6_H^f^, C_7_H^f^	7.35 (s), 7.69 (m), 7.19 (m), 7.27 (m), 7.49 (m)
Pyruvate^b^	CH_3_^e^	2.35 (s)
Azelate^c^	C_2,8_H_2_^f^, C_3,7_H_2_^e^_,_C_4,5,6_H_2_^e^	2.16 (t), 1.53 (m), 1.29 (m)
3-Methylxanthine^c^	CH^e^, CH_3_^e^	7.99 (s) , 3.52 (s)
Vanillate^c^	C_2_H^f^_,_ C_5_H^e^, C_6_H^e^, CH_3_^f^	7.53 (d), 6.94 (d), 7.45 (dd), 3.90 (s)
*N*-Methylhydantoin^b^	NCH_3_^f^, CH_2_^f^	2.92 (s), 4.06 (s)
5,6-Dihydrothymine^c^	CH_2_^e^_,_ CH_1_^f^, CH_3_^e^	3.33 (m), 2.78 (m), 1.20 (d)
3-Hydroxybutyrate^c^	C_2_H_2_^f^, C_3_H^f^, C_4_H_3_^e^	2.34 (m), 4.16 (m), 1.19 (d)
Acetone^b^	CH_3_^e^	2.21 (s)
Benzoate^b^	C_2,6_H^f^, C_3,5_H^f^, C_4_H^f^	7.88 (m), 7.49 (m), 7.56 (m)
Phenylacetate^c^	CH_2_^f^, C_2,6_H^f^, C_3,5_H^f^, C_4_H^f^	3.54 (s), 7.30 (m), 7.38 (m), 7.31 (m)
Thymine^c^	CH_3_^f^, CH^f^	1.86 (d), 7.37 (q)
Uracil^b^	NCH^f^, CCH^f^	7.55 (d), 5.80 (d)
Glycerol^c^	CH_3_^f^, CH_3_^f^,CH^f^	3.56 (dd), 3.66 (dd), 3.79 (m)
Propionate^c^	CH_3_^f^, CH_2_^f^	1.06 (t), 2.19 (q)
Urea^a^	NH_2_^f^	5.80 (s)
Fucose^c^	CH_3_^f^, C_1_H^f^, C_2_H^f^	1.21, 1.22, 1.25, 1.26 (s, s, s, s), 5.22, 4.53(d, d), 3.46 (dd)
4-Hydroxyphenylacetate^b^	CH_2_^f^, C_2,6_H^f^, C_3,5_H^f^	3.45 (s), 7.17 (m), 6.87 (m)
Creatinine^a^	CH_3_^f^, CH_2_^f^	3.04 (s), 4.05 (s)
Sarcosine^c^	CH_3_^f^, CH_2_^f^	2.73 (s), 3.60 (s)
Choline^b^	CH_3_^f^, NCH_2_^f^, OCH_2_^f^	3.21 (s), 3.53 (m), 4.07 (m)
Levulinate^c^	CH_3_^f^, C_3_H_2_^f^, C_2_H_2_^f^	2.23 (t), 2.78 (m), 2.41 (t)
Methylguanidine^b^	CH_3_^e^	2.83 (s)
1-Methylnicotinamide^a^	CH_3_^f^, C_2_H^f^, C_4_H^f^, C_5_H^f^, C_6_H^f^	4.49 (s), 9.29 (s), 8.91 (m), 8.19 (m), 8.97 (m)
3-Hydroxyisovalerate^b^	CH_3_^f^, CH_2_^f^	1.27 (s), 2.37 (s)
Fumarate^d^	CH^e^	6.50 (s)
Methylamine^b^	CH_3_^e^	2.61 (s)
Ethylmalonate^c^	CH_3_^f^, CH_2_^f^, CH^f^	0.89 (t), 1.72 (m), 2.99 (t)
4-Aminohippurate^c^	NH^e^, C_2,6_H^f^, C_3,5_H^f^, CH_2_^f^	8.20 (t), 7.66 (d), 6.88 (d), 3.92 (d)
Ethylene glycol^b^	CH_2_^e^	3.65 (s)
Ascorbate^c^	CH_2_^f^, CCH^f^, OCH^f^	3.74 (m), 4.03 (m), 4.53 (d)
Methylmalonate^c^	CH_3_^f^, CH^f^	1.24 (d), 3.17 (q)
Methylsuccinate^c^	CH_3_^f^, CH_2_^f^, CH^f^	1.09 (d),2.13, 2.52 (dd, dd) , 2.62 (m)
Malonate^b^	CH_2_^e^	3.12 (s)
Hypoxanthine^b^	C_2_H^e^, C_8_H^e^	8.17 (s), 8.19 (s)
*N*-Acetylglycine^c^	CH_3_^f^, CH_2_^e^	2.03 (s), 3.74 (d)
*N*-Phenylacetylglycine^b^	NCH_2_^f^, CH_2_^f^, C_2,6_H^f^, C_3,5_H^f^, C_4_H^f^	3.68 (s), 3.75 (s), 7.35 (m), 7.42 (m), 7.36 (m)
Hippurate^b^	CH_2_^f^, C_2,6_H^f^, C_3,5_H^f^, C_4_H^f^	3.97 (s), 7.56 (m), 7.84 (m), 7.64 (m)
Lactate^b^	CH_3_^f^, CH^f^	1.33 (d), 4.12 (q)
Xanthine^b^	CH^e^	7.86 (s)
*N*-Isovaleroylglycine^c^	CH_3_^f^, CH_2_^f^, CH^f^, NCH_2_^f^	0.94 (d), 2.18 (d), 2.01 (m), 3.76 (s)
Succinate^a^	CH_2_^e^	2.41 (s)
Glycolate^b^	CH_2_^e^	3.95 (s)
Formate^a^	CH^e^	8.46 (s)
Threonate^c^	C_2_H^f^, C_3_H^f^, CH_2_^f^	4.03 (d), 3.99 (m), 3.63, 3.70 (dd, dd)
Acetaminophen^c^	C_2,6_H_2_^f^, C_3,5_H_2_^e^, CH_3_^f^	6.90 (d), 7.22 (d), 2.13 (s)
Pantothenate^c^	CH_3_^f^, CH_2_^f^, CH^f^, NCH_2_^e^, COCH_2_^e^	0.90, 0.94 (s, s), 3.40, 3.52 (d, d), 3.99 (s), 3.43 (m), 2.42 (t)
Taurine^b^	NCH_2_^f^, SCH_2_^f^	3.28 (t), 3.43 (t)
*N*-Acetylglutamine^c^	CH_3_^f^, NCH^e^, C_4_H_2_^f^, C_3_H_2_^f^	2.04 (s), 4.15 (m), 2.33 (m), 1.93, 2.12 (m, m)
Pyroglutamate^c^	C_2_H_2_^f^, C_3_H_2_^e^, CH^f^	2.04, 2.51 (m, m), 2.41 (m), 4.18 (dd)
Betaine^b^	CH_3_^f^, CH_2_^f^	3.27 (s), 3.91 (s)
2-Oxoglutarate^a^	C_3_H_2_^f^, C_4_H_2_^f^	3.01 (t), 2.45 (t)
Pimelate^c^	CH_2_^f^, C_3,5_H_2_^e^, C_2,6_H_2_^e^	2.17 (t), 1.55 (m), 1.29 (m)
Acetamide^b^	CH_3_^e^	2.00 (s)
Trigonelline^a^	CH_3_^f^, C_2_H^f^, C_4_H^f^, C_5_H^f^, C_6_H^f^	4.44 (s), 9.13 (s), 8.84 (m), 8.09 (m), 8.84 (m)
*N*,*N*-Dimethylglycine^b^	CH_3_^f^, CH_2_^f^	2.93 (s), 3.73 (s)
Trimethylamine^d^	CH_3_^e^	2.89 (s)
1,3-Dimethylurate^c^	C_1_H_3_^e^, C_3_H_3_^e^	3.44 (s), 3.31 (s)
2-Hydroxyglutarate^c^	C_2_H^f^, C_3_H_2_^f^, C_4_H_2_^f^	4.03 (dd), 1.85, 2.00 (m, m), 2.25 (m)
2-Hydroxyisobutyrate^b^	CH_3_^e^	1.34 (s)
*cis*-Aconitate^a^	CH_2_^f^, CH^f^	3.13 (d), 5.73 (t)
Glutarate^c^	C_2,4_H_2_^f^, C_3_H_2_^f^	2.19 (m), 1.79 (m)
Valine^c^	CH_3_^f^	1.00, 1.05 (d, d)
Malate^c^	CH_2_^f^	2.68, 2.36 (dd, dd)
*N*-Acetylaspartate^c^	CH_2_^f^, C_2_H^e^, NH^e^, CH_3_^f^	2.49, 2.68 (dd), 4.38(dd), 7.91(d), 2.01(s)
Pipecolate^c^	C_2_H^f^, C_3_H_2_^f,e^, C_4_H_2_^f^, C_5_H_2_^f^, C_6_H_2_^f^	3.59 (dd), 1.67, 2.20 (m, m), 1.58, 1.89 (m, m), 1.65, 1.87(m, m), 3.01, 3.42(m, m)
Proline^c^	C_2_H^f^, C_3_H_2_^e^, C_4_H_2_^e^, C_5_H_2_^f^	4.14 (dd), 2.06, 2.35 (m, m), 1.98, 2.02 (m, m), 3.35, 3.42(m, m)
Tartrate^b^	CH^e^	4.34 (s)
*trans*-Aconitate^c^	CH_2_^f^, CH^f^	3.45 (d) , 6.59 (t)
Alanine^b^	CH_3_^f^, CH^f^	1.49 (d), 3.79 (q)
Creatine^b^	CH_3_^f^, CH_2_^f^	3.04 (s), 3.93 (s)
5,6-Dihydrouracil^c^	C_5_H_2_^e^, C_6_H_2_^e^	3.45(t) , 2.67 (t)
Glutamate^c^	C_2_H^f^, C_3_H_2_^f^, C_4_H_2_^f^	3.77 (dd), 2.10 (m), 2.35 (m)
Maltose^c^	C_1_H^e^, C_2_H^f^, C_7_H^f^	5.24 (d), 3.29, 3.55 (dd, m), 5.42 (d)
Glycine^a^	CH_2_^e^	3.57 (s)
4-Aminobutyrate^c^	C_2_H_2_^e^, C_3_H_2_^e^, C_4_H_2_^e^	2.29 (t), 1.89 (m), 3.01 (t)
Ethanolamine^c^	C_1_H_2_^f^, C_2_H_2_^e^	3.14 (d), 3.82 (d)
Citrate^a^	CH_2_^f^, CH_2_^f^	2.56 (d), 2.71 (d)
Guanidoacetate^b^	CH_2_^e^	3.80 (s)
TMAO^a^	CH_3_^e^	3.27 (s)
Sucrose^c^	C_2_H^f^, C_3_H^f^, C_5_H_2_^f^, C_6_H^f^, C_7_H^f^, C_8_H^f^	4.07 (t), 4.24 (d), 3.68, 3.69 (s, s), 5.44 (d), 3.58 (dd), 3.54 (t)

*TMAO* trimethylamine *N*-oxide, *s* singlet, *d* doublet, *t* triplet, *q* quartet, *dd* doublet of doublets, *m* multiplet

^a^Already clearly identified in the reference urine profile

^b^Clearer in fractions than in the reference urine profile

^c^Could not be seen in the reference urine profile

^d^Still not clear in fractions

^e^Putative assignment

^f^Positive assignment

**Table 2:** ^1^H NMR chemical shift assignment of metabolites in feces fractions

Metabolite	Assignment	*δ* ^1^H (ppm) and multiplicity
Ethanol^a^	CH_3_^d^, CH_2_^d^	1.19 (t), 3.66 (q)
Glycerol^a^	CH_3_^d^, CH_3_^d^, CH^d^	3.56 (dd), 3.65 (dd), 3.79 (m)
Lactate^a^	CH_3_^d^, CH^d^	1.33 (d), 4.12 (q)
Formate^a^	CH^e^	8.46 (s)
2-Hydroxyglutarate^b^	C_2_H^d^, C_3_H_2_^d^, C_4_H_2_^d^	4.03 (dd), 1.85, 2.00 (m, m), 2.26 (m)
Aspartate^b^	C_2_H^d^, C_3_H_2_^d^	3.90 (dd), 2.69, 2.81 (dd, dd)
Glutamate^a^	C_2_H^d^, C_3_H_2_^d^, C_4_H_2_^d^	3.77 (dd), 2.10 (m), 2.35 (m)
Glutarate^b^	C_2,4_H_2_^d^, C_3_H_2_^d^	2.19 (m), 1.79 (m)
Glycine^c^	CH_2_^d^	3.57 (s)
Lysine^b^	C_2_H^e^, C_3_H_2_^d^, C_4_H_2_^d^, C_5_H_2_^d^, C_6_H_2_^d^	3.77 (t), 1.92 (m), 1.48 (m), 1.74 (m), 3.03 (m)
Succinate^c^	CH_2_^d^	2.41(s)
Acetate^c^	CH_3_^e^	1.92 (s)
Alanine^c^	CH_3_^d^, CH^d^	1.49 (d), 3.79(q)
Glucose^a^	C_1_H^d^, C_2_H^d^, C_3_H^d^, C_4_H^d^, C_5_H^d^, C_6_H_2_^d^	4.66, 5.24 (d, d), 3.26, 3.55 (dd, dd), 3.50, 3.72 (t,t), 3.42 (m), 3.47, 3.83 (m, m), 3.74, 3.88 (m, m)
Proline^a^	C_2_H^d^, C_3_H_2_^e^, C_4_H_2_^e^, C_5_H_2_^d^	4.14 (dd), 2.06, 2.35 (m, m), 1.98, 2.02 (m, m), 3.35, 3.42 (m,m)
Mannitol^b^	C_1_H_2_^d^,C_6_H_2_^d^, C_2,5_H^e^, C_3,4_H^e^	3.90 (dd), 3.66 (dd), 3.75 (m), 3.79 (d)
Propionate^c^	CH_3_^d^, CH_2_^d^	1.06 (t), 2.19 (q)
Threonine^b^	C_2_H^e^, C_3_H^e^, C_4_H_3_^d^	3.58 (d), 4.26 (m), 1.34 (d)
2-Hydroxyisovalerate^b^	CH_3_^d^, C_2_H^d^, C_3_H^d^	0.84, 0.97 (d, d), 3.85 (d), 2.01 (m)
4-Hydroxybenzoate^b^	C_2,6_H^d^, C_3,5_H^d^	7.81 (m), 6.93 (m)
Butyrate^c^	CH_3_^d^, C_2_H_2_^d^, C_3_H_2_^d^	0.90(t), 2.16(t), 1.56 (m)
Choline^a^	CH_3_^d^, NCH_2_^d^, OCH_2_^e^	3.21(s), 3.52(m), 4.07(m)
Pimelate^b^	C_4_H_2_^d^, C_2,6_H_2_^e^, C_3,5_H_2_^e^	2.16 (t), 1.56 (m), 1.29 (m)
Fucose^b^	CH_3_^d^, C_1_H^d^	1.21, 1.22, 1.25, 1.26(s, s, s, s), 5.21, 4.56 (d,d)
Arginine^b^	C_2_H^e^, C_3_H_2_^e^, C_4_H_2_^e^, NH^e^, NH_2_^e^	3.25 (t), 1.92 (m), 1.64, 1.72 (m), 3.76(t), 3.25(t)
Malonate^a^	CH_2_^e^	3.12 (s)
Nicotinate^b^	C_2_H^d^, C_4_H^d^, C_5_H^d^, C_6_H^d^	8.95 (m), 8.26 (m), 7.53 (m), 8.60 (m)
Urocanate^b^	C_2_H^d^, C_5_H^d^, αCH^d^, βCH^d^	7.86 (s), 7.40 (s), 6.40 (d), 7.31 (d)
Valine^a^	CH_3_^d^, C_2_H^d^, C_3_H^d^	1.00, 1.05 (d, d), 3.62(d), 2.28(m)
Methionine^a^	C_2_H^d^, C_3_H_2_^d^, C_4_H_2_^d^, CH_3_^d^	3.86 (dd), 2.11, 2.19 (m, m), 2.65(t), 2.14(s)
Pyruvate^a^	CH_3_^e^	2.38 (s)
2-Hydroxy-3-methylvalerate^b^	C_2_H^d^, C_3_H^e^, C_4_H_2_^d^, C_3_CH_3_^d^, C_5_H_3_^d^	3.88 (d), 1.75 (m), 1.17, 1.36 (m, m), 0.93 (d), 0.88 (t)
2-Hydroxyisocaproate^b^	C_2_H^d^, C_3_H_2_^d^, C_4_H^d^, CH_3_^d^, CH_3_^d^	4.05 (dd), 1.52 (m), 1.75 (m), 0.93 (d), 0.93 (d)
4-Hydroxyphenylacetate^b^	CH_2_^d^, C_2,6_H^d^, C_3,5_H^d^	3.45 (s), 7.17 (m), 6.87 (m)
Creatinine^a^	CH_3_^d^, CH_2_^d^	3.05 (s), 4.06 (s)
Dimethylamine^a^	CH_3_^e^	2.71 (s)
Methylamine^a^	CH_3_^e^	2.60 (s)
Taurine^b^	NCH_2_^d^, SCH_2_^d^	3.25 (t), 3.45 (t)
Alloisoleucine^b^	C_2_H^d^, C_3_H^d^, C_4_H_2_^d^, C_3_CH_3_^d^, C_5_H_3_^d^	3.74 (d), 2.07 (m), 1.35, 1.45 (m, m), 0.95 (d), 0.97 (t)
Isovalerate^b^	CH_2_^d^, CH^e^, CH_3_^e^	2.04 (d), 1.94 (m), 0.90 (d)
Benzoate^a^	C_2,6_H^d^, C_3,5_H^d^, C_4_H^d^	7.88 (m), 7.49 (m), 7.56 (m)
Isoleucine^b^	C_2_H^d^, C_3_H^d^, C_4_H_2_^d^, C_3_CH_3_^d^, C_5_H_3_^d^	3.68 (d), 1.99 (m), 1.27, 1.48 (m, m), 1.01 (d), 0.94 (t)
Leucine^a^	C_2_H^d^, C_3_H_2_^d^, C_4_H^d^, CH_3_^d^, CH_3_^d^	3.74 (m), 1.69, 1.75 (m, m), 1.72 (m), 0.96 (d), 0.97 (d)
Suberate^b^	C_2,7_H_2_^d^, C_3,6_H_2_^e^, C_4,5_H_2_^e^	2.18 (t), 1.55 (m), 1.30 (m)
Trimethylamine^a^	CH_3_^e^	2.89 (s)
Uracil^b^	C_5_H^d^, C_6_H^d^	5.81 (d), 7.55 (d)
3-Hydroxyphenylacetate^b^	CH_2_^d^, C_2_H^d^, C_4_H^d^, C_5_H^d^, C_6_H^d^	3.48 (s), 6.81 (m), 6.80 (m), 7.26 (t), 6.86 (m)
1,3-Dihydroxyacetone^b^	CH_2_^e^, CH_2_^e^	3.57 (s),4.41 (s)
2-Hydroxybutyrate^b^	CH^e^, CH_2_^e^, CH_3_^d^	3.99 (dd), 1.69 (m), 0.89 (t)
2-Oxoisocaproate^b^	C_3_H_2_^d^, C_4_H^d^, CH_3_^d^	2.62 (d), 2.10 (m), 0.94 (d)
3-Methyl-2-oxovalerate^b^	C_3_H^d^, C_4_H_2_^d^, C_3_CH_3_^d^, C_5_H_3_^d^	2.94 (m), 1.46, 1.71 (m, m), 1.10 (d), 0.90 (t)
Acetamide^a^	CH_3_^e^	2.01 (s)
Glycolate^a^	CH_2_^e^	3.92 (s)
Tyrosine^b^	C_2,6_H^d^, C_3,5_H^d^, αCH^d^, βCH^d^	7.20 (m), 6.91 (m), 3.95 (dd), 3.07, 3.20 (dd, dd)
Isobutyrate^b^	CH_3_^d^, CH^e^	1.05 (d), 2.38 (m)
*N*-Methylhydantoin^a^	NCH_3_^d^, CH_2_^d^	2.93(s), 4.09(s)
Phenylacetate^b^	CH_2_^d^, C_2,6_H^d^, C_3,5_H^d^, C_4_H^d^	3.54 (s), 7.31 (m), 7.39 (m), 7.31 (m)
3-Phenyllactate^b^	C_2,6_H^d^, C_3,5_H^d^, C_4_H^d^, αCH^d^, βCH_2_^d^	7.32 (m), 7.38 (m), 7.31 (m), 4.27 (dd), 2.89, 3.11 (dd, dd)
2-Hydroxyphenylacetate^b^	C_3,5_H^d^,C_4,6_H^e^, CH_2_^d^	7.19 (m), 6.91 (m), 3.54 (s)
Guanidoacetate^a^	CH_2_^e^	3.80 (s)
2-Hydroxyvalerate^b^	C_2_H^e^, C_3_H_2_^e^, C_4_H_2_^e^, C_5_H_3_^e^	4.06 (dd), 1.61, 1.69 (m, m), 1.35 (m), 0.89 (t)
*N*-Acetylornithine^b^	CH_3_^d^, C_2_H^e^, C_3_H_2_^e^, C_4_H_2_^e^, C_5_H_2_^e^	2.04 (s), 4.17 (m), 1.75, 1.84 (m, m), 1.69 (m), 3.01 (m)
Isovalerate^b^	CH_3_^e^, CH_2_^e^, CH^e^	0.91 (d), 2.05 (d), 1.95 (m)
Uridine^b^	C_5_H^d^,C_6_H^d^,C_7_H^d^, C_8_H^d^, C_9_H^d^	5.91 (d), 7.88 (d), 5.93 (d), 4.36 (t), 4.24 (t)
Thymine^b^	CH_3_^d^, CH^d^	1.87 (s), 7.38 (s)
Homovanillate^b^	C_2_H^d^, C_4_H^d^, C_5_H^d^, CH_2_^d^, OCH_3_^d^	6.97 (d), 6.80 (dd), 6.88 (d), 3.36 (s), 3.87 (s)
Phenylalanine^b^	C_2,6_H^d^, C_3,5_H^d^, C_4_H^d^, αCH^d^, βCH_2_^d^	7.34 (m), 7.43 (m), 7.38 (m), 4.00 (dd), 3.14, 3.28 (dd, dd)
2'-Deoxyadenosine^b^	C_1′_H^e^, C_2′_H_2_^e^, C_3′_H^e^, C_4′_H^e^, C_8_H^d^, C_3_H^d^	6.50 (t), 2.56, 2.85 (m, m), 4.66 (m), 4.18 (m), 8.33 (s), 8.24 (s)
3-Phenylpropionate^b^	C_2,6_H^d^, C_3,5_H^d^, C_4_H^e^, αCH_2_^d^, βCH_2_^d^	7.32 (m), 7.37 (m), 7.27 (m), 2.49 (t), 2.88 (m)
Anserine^b^	C_2_H^e^, C_5_H^d^, αCH^e^, βCH_2_^e^, NCH_2_^e^, COCH_2_^e^	8.24 (s), 7.12 (s), 4.44 (m), 3.04, 3.20 (dd, dd), 2.72 (m), 3.20 (m)
Thymidine^b^	C_5_CH_3_^d^, C_6_H^d^, C_1′_H^d^, C_2′_H_2_^d^, C_3′_H^d^, C_4′_H^d^, C_5′_H_2_^e^	1.90 (d), 7.65 (m), 6.31 (t), 2.38 (dd), 4.48 (m), 4.03 (m), 3.78, 3.85 (dd, dd)
Tryptophan^b^	C_2_H^d^, C_6_H^d^, C_7_H^d^, C_8_H^d^, C_9_H^d^, βCH_2_^d^,αCH^d^	7.34 (s), 7.74 (m), 7.20 (m), 7.29 (m), 7.55 (m), 3.32, 3.49 (dd, dd), 4.06 (dd)
Tyramine^b^	C_2,6_H^d^, C_3,5_H^d^, αCH_2_^d^, βCH_2_^d^	7.23 (m), 6.92 (m), 2.93(t), 3.24 (t)

*s* singlet, *d* doublet, *t* triplet, *q* quartet, *dd* doublet of doublets, *m* multiplet

^a^Clearer in fractions than in the reference urine profile

^b^Could not be seen in the reference urine profile

^c^Already clear in the reference urine profile

^d^Positive assignment

^e^Putative assignment

**Fig. 3:**
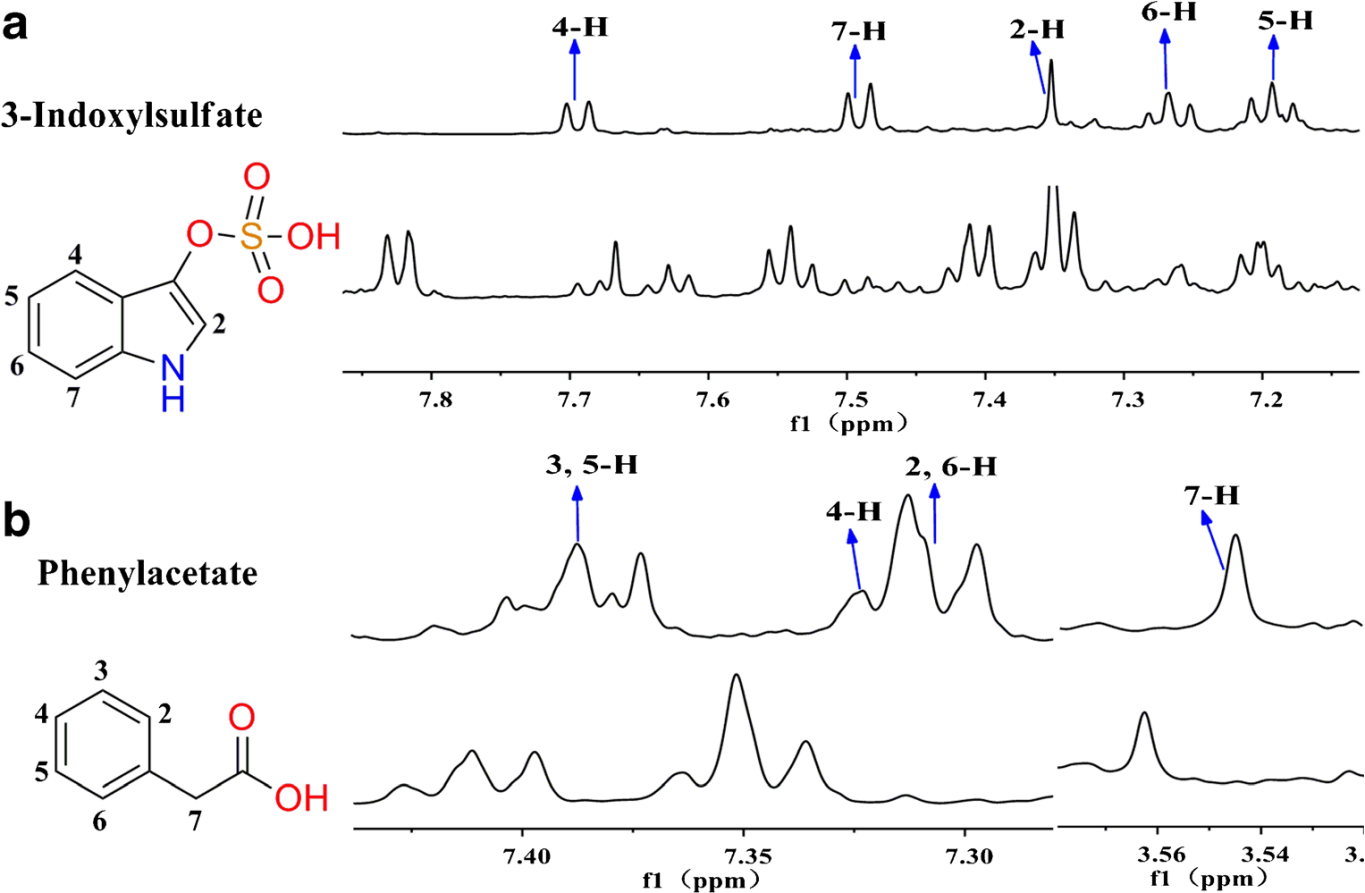
Comparison of urine metabolite identifications from ^1^H NMR spectra of high-performance liquid chromatography (HPLC) fractions (*upper spectra*) and from the reference profile (*lower spectra*)

**Fig. 4:**
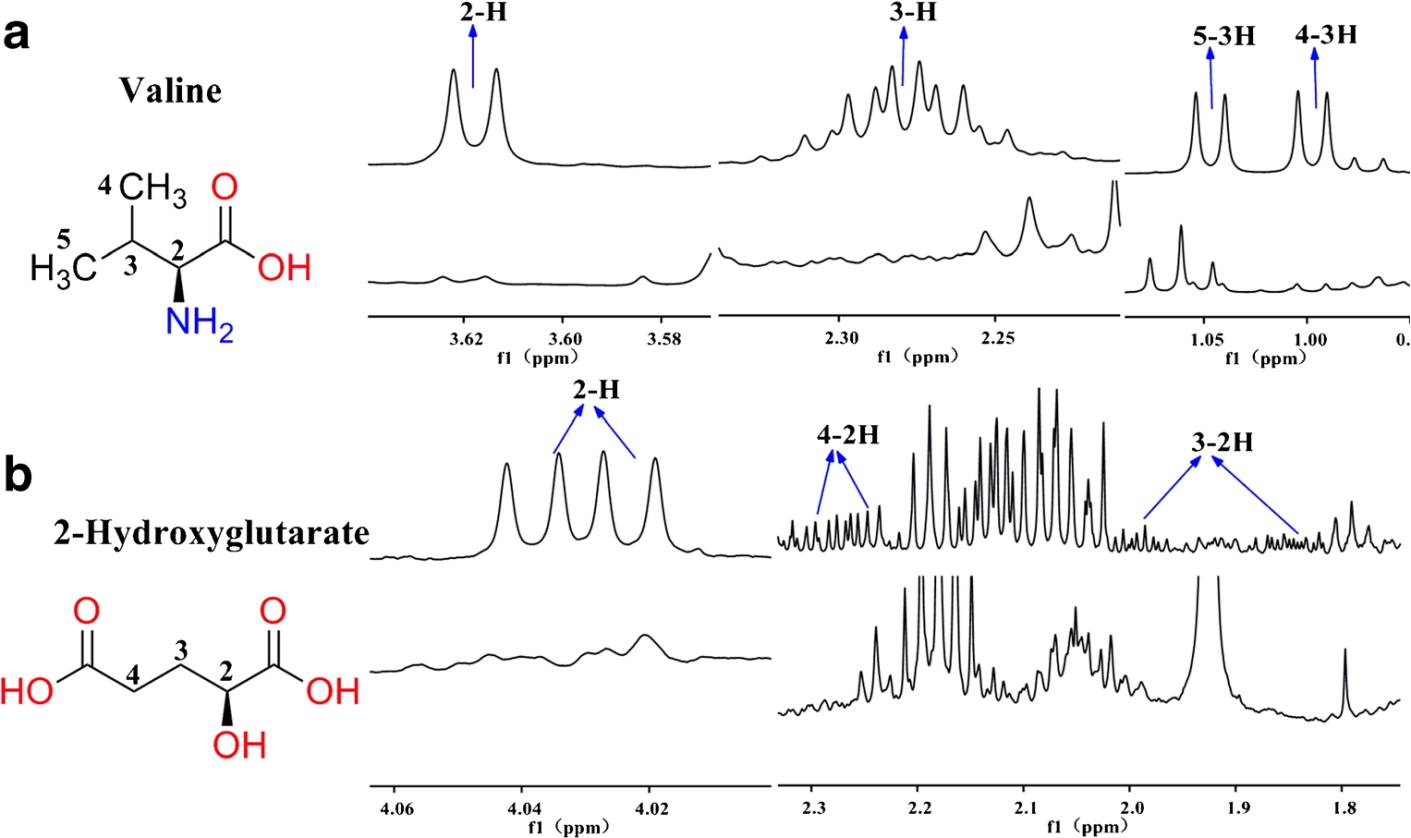
Comparison of feces metabolite identifications from ^1^H NMR spectra of HPLC fractions (*upper spectra*) and from the reference profile (*lower spectra*)

With the HPLC enrichment methods, metabolites could be clearly recognized, as shown in Figs. 3a and 4a. Moreover, some trace amounts of metabolites could be clearly observed, as shown in Figs. 3b and 4b. In each figure, the upper spectra are the measurement results of the HPLC fractions, and the lower spectra show the same chemical shift range from ^1^H NOESY NMR spectra of reference urine and feces samples. Here 3-indoxyl sulfate and valine are used as examples to explain how our method worked. In Fig. 3a, 3-indoxyl sulfate shows strong resonances without interfering signals from other metabolites in the urine fraction. But it could not be clearly identified in the reference urine profile, owing to the overlapped resonances. The result for valine was the same as shown in Fig. 4a. Valine had apparent characteristic peaks in the HPLC fraction, but the signals were very weak in the reference feces profile because 3-H could not be clearly identified. Phenylacetate and 2-hydroxyglutarate are used as examples to explain how trace amounts of metabolites could also be clearly identified using this method. In Fig. 3b, phenylacetate has very clear resonances in the HPLC urine fractions, but shows no signal in the reference urine profile. Figure 4b shows that 2-hydroxyglutarate had obvious characteristic signals in the HPLC feces fractions, but not in the reference feces profile.

**Fig. 5:**
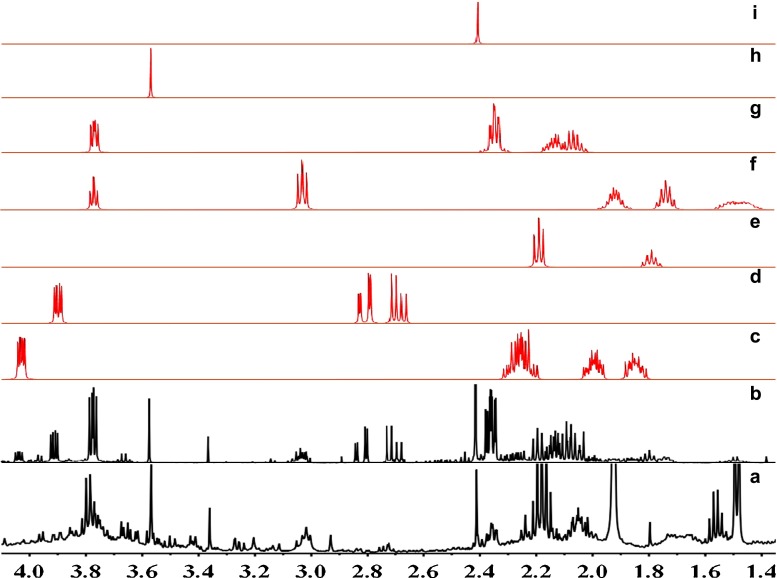
Typical comparison between the reference sample, fraction 3, and standard substances (from Chenomx NMR Suite 7.7 Library Manager). *A* reference sample, *B* fraction 3, *C* 2-hydroxyglutarate, *D* aspartate, *E* glutarate, *F* lysine, *G* glutamate, *H* glycine, *I* succinate

**Fig. 6:**
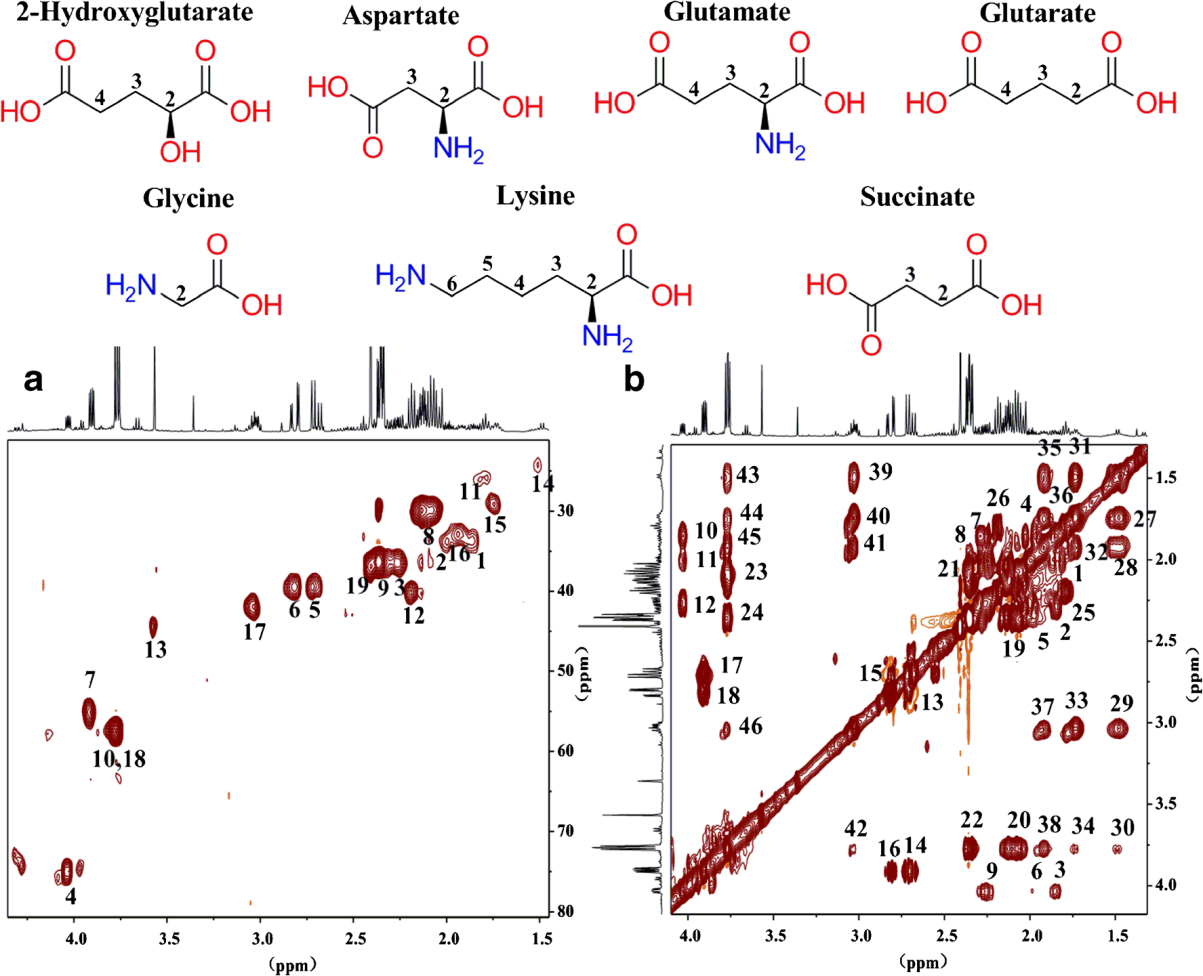
Typical heteronuclear single quantum correlation (*A*) and total correlation spectroscopy (*B*) spectra of the feces HPLC (C_18_) fraction. For spectrum A, *1*, *2* 2-hydroxyglutarate (3-CH_2_), *3* 2-hydroxyglutarate (4-CH_2_), *4* 2-hydroxyglutarate (2-CH), *5*, *6* aspartate (3-CH_2_), *7* aspartate (2-CH), *8* glutamate (3-CH_2_), *9* glutamate (4-CH_2_), *10* glutamate (2-CH), *11* glutarate (3-CH_2_), *12* glutarate (2,4-CH_2_), *13* glycine (2-CH_2_), *14* lysine (4-CH_2_), *15* lysine (5-CH_2_), *16* lysine (3-CH_2_), *17* lysine (6-CH_2_), *18* lysine (2-CH), *19* succinate (2,3-CH_2_). For spectrum B, *1*, *4* 2-hydroxyglutarate (3H–3H), *2*, *5* 2-hydroxyglutarate (3H–4H), *3*, *6* 2-hydroxyglutarate (3H–2H), *7*, *8* 2-hydroxyglutarate (4H–3H), *9* 2-hydroxyglutarate (4H–2H), *10*, *11* 2-hydroxyglutarate (2H–3H), *12* 2-hydroxyglutarate (2H–4H), *13*, *15* aspartate (3H–3H), *14*, *16* aspartate (3H–2H), *17*, *18* aspartate (2H–3H), *19* glutamate (3H–4H), *20* glutamate (3H–2H), *21* glutamate (4H–3H), *22* glutamate (4H–2H), *23* glutamate (2H–3H), *24* glutamate (2H–4H), *25* glutarate (3H–2,4H), *26* glutarate (2,4H–3H), *27* lysine (4H–5H), *28* lysine (4H–3H), *29* lysine (4H–6H), *30* lysine (4H–2H), 31 lysine (5H–4H), *32* lysine (5H–3H), *33* lysine (5H–6H), *34* lysine (5H–2H), *35* lysine (3H–4H), *36* lysine (3H–5H), *37* lysine (3H–6H), *38* lysine (3H–2H), *39* lysine (6H–4H), *40* lysine (6H–5H), 41 lysine (6H–3H), *42* lysine (6H–2H), *43* lysine (2H–4H), *44* lysine (2H–5H), *45* lysine (2H–3H), *46* lysine (2H–6H)

In complex biological systems,there are many similar structures or groups of metabolites, called homologues or derivatives, such as the seven metabolites shown in Fig. 5. Their signals are very dense between 2.0 and 4.0 ppm in ^1^H NMR spectra. Owing to changes of chemical shifts under different environments, it is difficult to identify the corresponding peaks in the reference spectrum (Fig. 5, spectrum A). HPLC provides a better characterization method (Fig. 5, spectrum B). Among the fractions, 2-hydroxyglutarate (Fig. 5, spectrum C), aspartate (Fig. 5, spectrum D), glutarate (Fig. 5, spectrum E), and lysine (Fig. 5, spectrum F) could be recognized, whereas glutamate (Fig. 5, spectrum G) was better recognized in the fraction than in the reference feces profile. Further experiments were performed using the combined techniques of HPLC and two-dimensional HSQC or TOCSY NMR spectroscopy to verify metabolite identities. All ^1^H–^13^C single bond signals and ^1^H–^1^H totally correlated signals are shown clearly in Fig. 6. Both glycine (Fig. 5, spectrum H) and succinate (Fig. 5, spectrum I) had one single resonance in the ^1^H NMR spectrum, and in Fig. 6, spectrum A, their ^1^H–^13^C single-bond correlated signals are further identified (Table 3).

**Table 3:** The NMR signal assignment of metabolites in a feces fraction

Metabolite	*δ* ^1^H (ppm)	HSQC *δ* ^1^H–^13^C (ppm)	TOCSY *δ* ^1^H–^1^H (ppm)
2-Hydroxyglutarate	1.85	1.85, 33.55	1.86, 2.00; 1.86, 2.27; 1.85, 4.03
	2.00	1.99, 33.55	2.01, 1.85; 2.00, 2.26; 1.99, 4.03
	2.26	2.25, 36.35	2.27, 1.85; 2.25, 2.01; 2.26, 4.03
	4.03	4.03, 74.94	4.03, 1.86; 4.03, 2.01; 4.03, 2.26
Aspartate	2.69	2.71, 39.35	2.69, 2.81; 2.69, 3.91
	2.81	2.80, 39.34	2.82, 2.69; 2.80, 3.91
	3.90	3.91, 54.99	3.91, 2.70; 3.91, 2.81
Glutamate	2.10	2.11, 29.79	2.10, 2.36; 2.10, 3.78
	2.35	2.35, 36.22	2.35, 2.10; 2.35, 3.78
	3.77	3.77, 57.35	3.77, 2.10; 3.78, 2.36
Glutarate	1.79	1.78, 25.77	1.80, 2.20
	2.19	2.18, 40.08	2.20, 1.80
Glycine	3.57	3.57, 44.32	No signal
Lysine	1.48	1.48, 24.50	1.48, 1.74; 1.48, 1.92; 1.48, 3.03; 1.48, 3.77
	1.74	1.74, 29.11	1.74, 1.48; 1.74, 1.92; 1.74, 3.03; 1.74, 3.77
	1.92	1.92, 32.70	1.92, 1.48; 1.92, 1.74; 1.92, 3.03; 1.92, 3.77
	3.03	3.03, 41.82	3.03, 1.48; 3.03, 1.74; 3.03, 1.92; 3.03, 3.77
	3.77	3.77, 57.35	3.77, 1.48; 3.77, 1.74; 3.77, 1.92; 3.77, 3.03
Succinate	2.41	2.40, 36.97	No signal

*HSQC* heteronuclear single quantum correlation, *TOSCY* total correlation spectroscopy

## Discussion

Jacobs et al. [36] previously applied SPE–NMR-based metabolomics during nutritional intervention trials. Their study proved that SPE–NMR-based metabolite subprofiling was a reliable and improved method, compared with the NMR approach, for metabolite identification in urine. In their experiments, an SPE column was used to separate each urine sample into three fractions to achieve more accurate metabolite quantification and identification, especially for the metabolites with low concentrations. In our experiment, HILIC and C_18_ analytical columns were applied to obtain improved metabolite subprofiling. The 1.7-mm microprobe can reduce the sample volume to one tenth of that for an ordinary probe, and contacted well with samples we collected from the liquid-phase analysis. Therefore, our method could significantly shorten the time for sample separation, enrichment, concentration, and collection, and reduce the number of animals required. In this research, we needed only 10 h to complete the sample separation and enrichment. In addition, it took only a few minutes to collect ^1^H NMR spectra for each collected sample fraction and a few hours to collect two-dimensional spectra for that fraction.

To ensure that fractions and the original sample were measured under the same neutral or weak alkaline condition, so that the chemical shifts of the two spectra are comparable, after freeze-drying, the separated fractions were dissolved in a buffer which had the same saline ratio as the buffer for the control. NMR experiments showed that the chemical shifts of metabolites in both spectra were similar (Fig. 5, spectra A and B), suggesting that the results were valid.

Among the 83 metabolites identified from rat urine, 20 had very simple NMR spectra with only a single resonance, such as an acetate or dimethylamine peak. To improve identification of those 20 metabolites, the interfering peaks from other metabolites must be removed. Therefore, it was necessary to separate those metabolites using analytical columns. Some metabolites might have overlapping resonances or have concentrations below the NMR detection limit in the reference urine sample. With the separation and enrichment using the HPLC method, those metabolites were identified in the HPLC fractions. Among the 40 metabolites only identified in fractions, 25 were positively identified, because of their complex characteristic or their strong resonances. The other 15 metabolites were putatively identified, such as *N*-acetylglycine, 1,3-dimethylurate, and proline. *N*-Acetylglycine had a simple spectrum with a single resonance and a doublet at about 2.03 and 3.74 ppm. It was putatively recognized, because the resonance at 2.03 ppm was clear but the 3.74-ppm resonance was obscured. 1,3-Dimethylurate had only two single resonances at 3.44 and 3.31 ppm. Since the two resonances were weak and close to other resonances, 1,3-dimethylurate could only be putatively identified. Proline had six groups of multiple resonances and one doublet of doublets. Among them, only the doublet of doublets at 4.14 ppm and one multiplet at 3.35 ppm could be positively identified. The other five multiplets were covered by other resonances. Proline could therefore only be putatively identified.

Among the 45 metabolites discovered in only feces fractions, 29 were positively identified, and the remaining 16 were putatively identified. All resonances of the 29 positively identified metabolites were clear and obvious, except for those of lysine, 2-hydroxy-3-methylvalerate, 3-phenylpropionate, and thymidine. Those four compounds all had complex spectra; however, most of their characteristic resonances were clear. Therefore, they were classified as positively identified. Fucose had four singlets at 1.21, 1.22, 1.25, and 1.26 ppm, two doublets, at 5.21 and 4.56 ppm, and several between 3.40 and 4.20 ppm. However, only four single peaks and two double peaks were positively identified. The other peaks were covered by other resonances. Fucose was therefore only putatively identified. And it was the same for the other 15 compounds.

Compound identification is an important and difficult task in metabolomics research. The accuracy of metabolite identification directly affects the results of metabolomic biological analysis. Some metabolites are important biomarkers for certain diseases, but they are often ignored owing to their low concentration and difficulty of identification. Urine and feces have been widely used as samples in many metabolomic studies, because of their relative ease of collection and low protein contents. Here, phenylacetate and 2-hydroxyglutarate are used as examples to elucidate how our methods worked. Both chemicals are present at very low concentrations in urine, and could be identified only through a fractional enrichment method. Plasma phenylacetate has been analyzed in patients with urination disorders or hepatic encephalopathy [37]. However, it has not been identified and quantified in urine or feces profiles. With use of our methods, urine and feces phenylacetate could be measured for the analysis of these diseases. The metabolic profiles of feces, plasma, and tumor tissue could be very useful in colorectal cancer diagnosis and treatment [38]. High levels of 2-hydroxyglutarate have been reported in both tumor tissues and plasma, but have not been found in urine and feces because of the limitation of the previous analytical methods. In our research, 2-hydroxyglutarate could be clearly identified in rat urine and feces. Therefore, with our method, colorectal cancer could be diagnosed much more conveniently using urine and feces samples.

## Conclusion

For NMR-based metabolomics research, the identification of some metabolites remains a big challenge owing to low abundance or strong signal overlaps. In this study, NMR spectroscopy combined with HPLC was applied to identify metabolites in complex biological mixtures. With this method, 83 and 73 metabolites were identified in Sprague Dawley rat urine and feces, respectively. We believe that more metabolites could be accurately identified by changing the chromatographic development conditions, using different columns, or changing the fraction collection time. Our research revealed that the coupling of HPLC and microprobe NMR spectroscopy techniques could improve metabolite identification, and is an effective and convenient approach to recognize biomarkers in complex biological systems.

At the same time, we also noticed that there were many visible peaks in the fragment spectra, but their structures could not be confirmed. Further work will be performed using the coupled HPLC, NMR spectroscopy, and mass spectrometry analysis method to characterize the structures from those peaks.

## Electronic supplementary material

Below is the link to the electronic supplementary material.

ESM 1 (PDF 1654 kb)

## References

[1] Clayton TA, Lindon JC, Cloarec O, Antti H, Charuel C, Hanton G, Provost JP, Le Net JL, Baker D, Walley RJ, Everett JR, Nicholson JK (2006). Nature.

[2] Zheng P, Gao HC, Li Q, Shao WH, Zhang ML, Cheng K, de Yang Y, Fan SH, Chen L, Fang L, Xie P (2012). J Proteome Res.

[3] Sun LY, Hu WH, Liu Q, Hao QF, Sun B, Zhang Q, Mao S, Qiao J, Yan XZ (2012). J Proteome Res.

[4] Figueroa JD, Cordero K, Serrano-Illan M, Almeyda A, Baldeosingh K, Almaguel FG, Leon M (2013). Neuroscience.

[5] Zira A, Kostidis S, Theocharis S, Sigala F, Engelsen SB, Andreadou I, Mikros E (2013). Toxicology.

[6] Astarita G, Langridge J (2013). J Nutrigenet Nutrigenomics.

[7] Wijeyesekera A, Selman C, Barton RH, Holmes E, Nicholson JK, Withers DJ (2012). J Proteome Res.

[8] Chen Y, Shen G, Zhang R, He J, Zhang Y, Xu J, Yang W, Chen X, Song Y, Abliz Z (2013). Anal Chem.

[9] Zhang H, Wu L, Xu C, Xia C, Sun L, Shu S (2013). BMC Vet Res.

[10] Appiah-Amponsah E, Shanaiah N, Nagana Gowda GA, Owusu-Sarfo K, Ye T, Raftery D (2009). J Pharm Biomed Anal.

[11] Fan WMT (1996). Prog Nucl Magn Reson Spectrosc.

[12] Bollard ME, Garrod S, Holmes E, Lindon JC, Humpfer E, Spraul M, Nicholson JK (2000). Magn Reson Med.

[13] Cui Q, Lewis IA, Hegeman AD, Anderson ME, Li J, Schulte CF, Westler WM, Eghbalnia HR, Sussman MR, Markley JL (2008). Nat Biotechnol.

[14] Wishart DS, Jewison T, Guo AC, Wilson M, Knox C, Liu Y, Djoumbou Y, Mandal R, Aziat F, Dong E, Bouatra S, Sinelnikov I, Arndt D, Xia J, Liu P, Yallou F, Bjorndahl T, Perez-Pineiro R, Eisner R, Allen F, Neveu V, Greiner R, Scalbert A (2013). Nucleic Acids Res.

[15] Jiang CY, Yang KM, Yang L, Miao ZX, Wang YH, Zhu HB (2013). PLoS One.

[16] Bouatra S, Aziat F, Mandal R, Guo AC, Wilson MR, Knox C, Bjorndahl TC, Krishnamurthy R, Saleem F, Liu P, Dame ZT, Poelzer J, Huynh J, Yallou FS, Psychogios N, Dong E, Bogumil R, Roehring C, Wishart DS (2013). PLoS One.

[17] Aue WP, Karhan J, Ernst RR (1976). J Chem Phys.

[18] Bax A, Freeman R (1981). J Magn Reson.

[19] Bax A, Davis DG (1985). J Magn Reson.

[20] Kay LE, Keifer P, Saarinen T (1992). J Am Chem Soc.

[21] Bax A, Summers MF (1986). J Am Chem Soc.

[22] Forseth RR, Schroeder FC (2011). Curr Opin Chem Biol.

[23] Liu H, Zheng A, Liu H, Yu H, Wu X, Xiao C, Dai H, Hao F, Zhang L, Wang Y, Tang H (2012). J Agric Food Chem.

[24] Lambert M, Wolfender JL, Staerk D, Christensen SB, Hostettmann K, Jaroszewski JW (2007). Anal Chem.

[25] Timmers MA, Dias DA, Urban S (2012). Mar Drugs.

[26] Johansen KT, Wubshet SG, Nyberg NT (2013). Anal Chem.

[27] Rezzi S, Vera FA, Martin FP, Wang S, Lawler D, Kochhar S (2008). J Chromatogr B.

[28] Akira K, Mitome H, Imachi M, Shida Y, Miyaoka H, Hashimoto T (2010). J Pharm Biomed Anal.

[29] Aranibar N, Vassallo JD, Rathmacher J, Stryker S, Zhang Y, Dai J, Janovitz EB, Robertson D, Reily M, Lowe-Krentz L, Lehman-McKeeman L (2011). Anal Biochem.

[30] Grimes JH, Conne TMO (2011). J Biomol NMR.

[31] Schütz C, Quitschau M, Hamburger M, Potterat O (2011). Fitoterapia.

[32] Spagou K, Wilson ID, Masson P, Theodoridis G, Raikos N, Coen M, Holmes E, Lindon JC, Plumb RS, Nicholson JK, Want EJ (2011). Anal Chem.

[33] Luo HG, Chen JX, Zhang Q, Yue GX, Ding J, Zhang HT, Yan XZ, Zhao X, Meng ZZ (2013). Chin J Integr Med.

[34] He CC, Dai YQ, Hui RR, Hua J, Chen HJ, Luo QY, Li JX (2012). J Appl Toxicol.

[35] Jiang H, Peng J, Zhou ZY, Duan Y, Chen W, Cai B, Yang H, Zhang W (2010). Chin Med J.

[36] Jacobs DM, Spiesser L, Garnier M, de Roo N, van Dorsten F, Hollebrands B, van Velzen E, Draijer R, van Duynhoven J (2012). Anal Bioanal Chem.

[37] Mokhtarani M, Diaz GA, Rhead W, Berry SA, Lichter-Konecki U, Feigenbaum A, Schulze A, Longo N, Bartley J, Berquist W, Gallagher R, Smith W, McCandless SE, Harding C, Rockey DC, Vierling JM, Mantry P, Ghabril M, Brown RS, Dickinson K, Moors T, Norris C, Coakley D, Milikien DA, Nagamani SC, Lemons C, Lee B, Scharschmidt BF (2013). Mol Genet Metab.

[38] Montrose DC, Zhou XK, Kopelovich L, Yantiss RK, Karoly ED, Subbaramaiah K, Dannenberg AJ (2012). Cancer Prev Res.

