# Supplementary material for: The application of HPLC and microprobe NMR spectroscopy in the identification of metabolites in complex biological matrices
Source: Anal Bioanal Chem. 2015 Mar 27;407(12):3405–16. doi: 10.1007/s00216-015-8556-y (PMC4392169; doi:10.1007/s00216-015-8556-y)
Supplement: Supplementary file 1 — (PDF 1654 kb) [file 216_2015_8556_MOESM1_ESM.pdf]

Analytical and Bioanalytical Chemistry

Electronic Supplementary Material

The application of HPLC and microprobe NMR spectroscopy in the identification of metabolites in complex biological matrices

Zhaoxia Miao, Mengxia Jin, Xia Liu, Wei Guo, Xiangju Jing, Hongyue Liu, Yinghong Wang

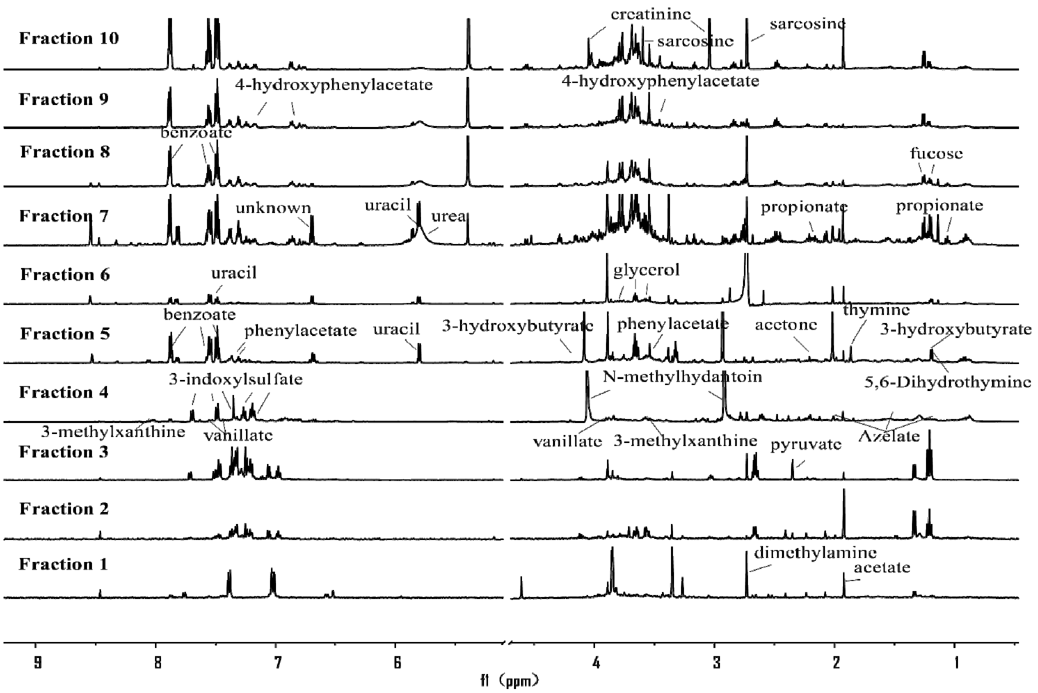

Fig. S1 NMR compositional analysis of rat urine HPLC fractions 1-10

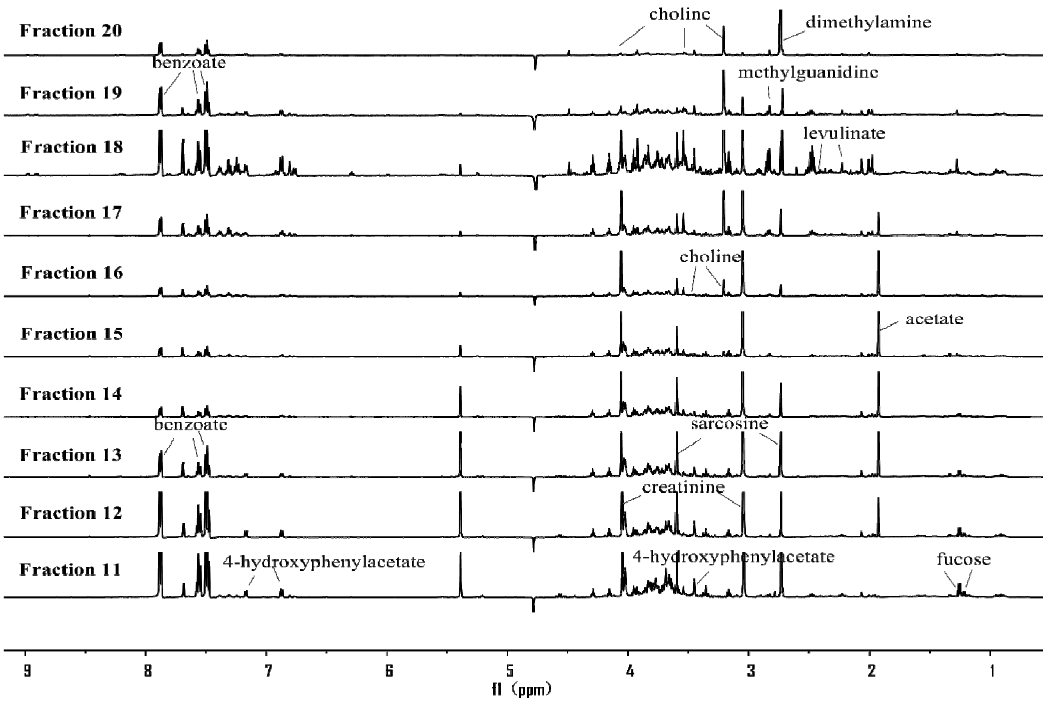

Fig. S2 NMR compositional analysis of rat urine HPLC fractions 11-20

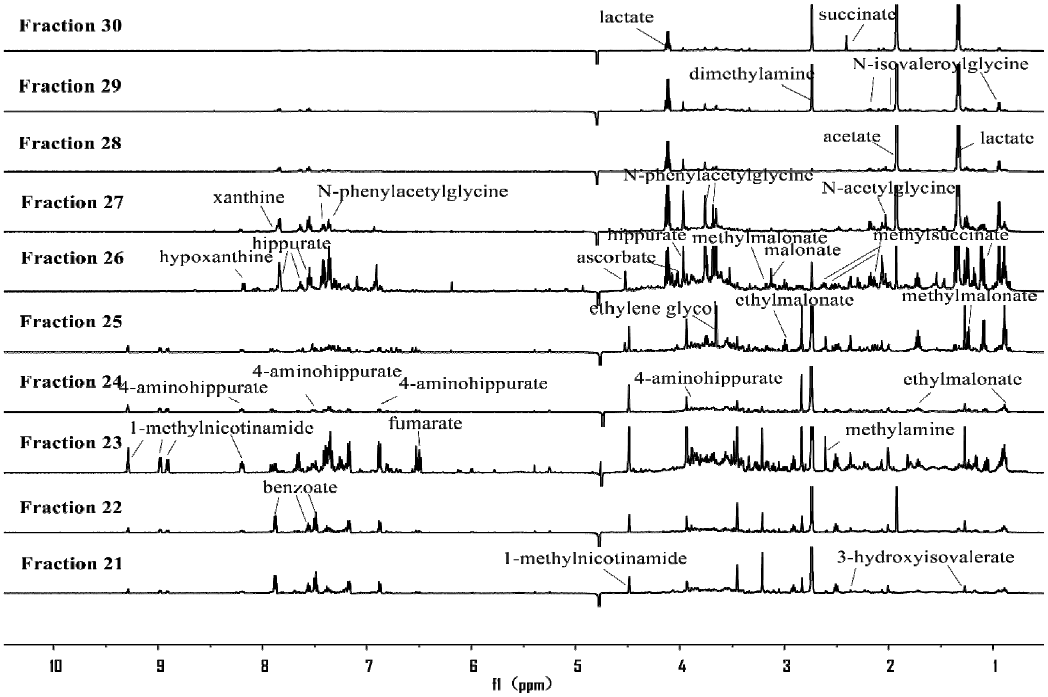

Fig. S3 NMR compositional analysis of rat urine HPLC fractions 21-30

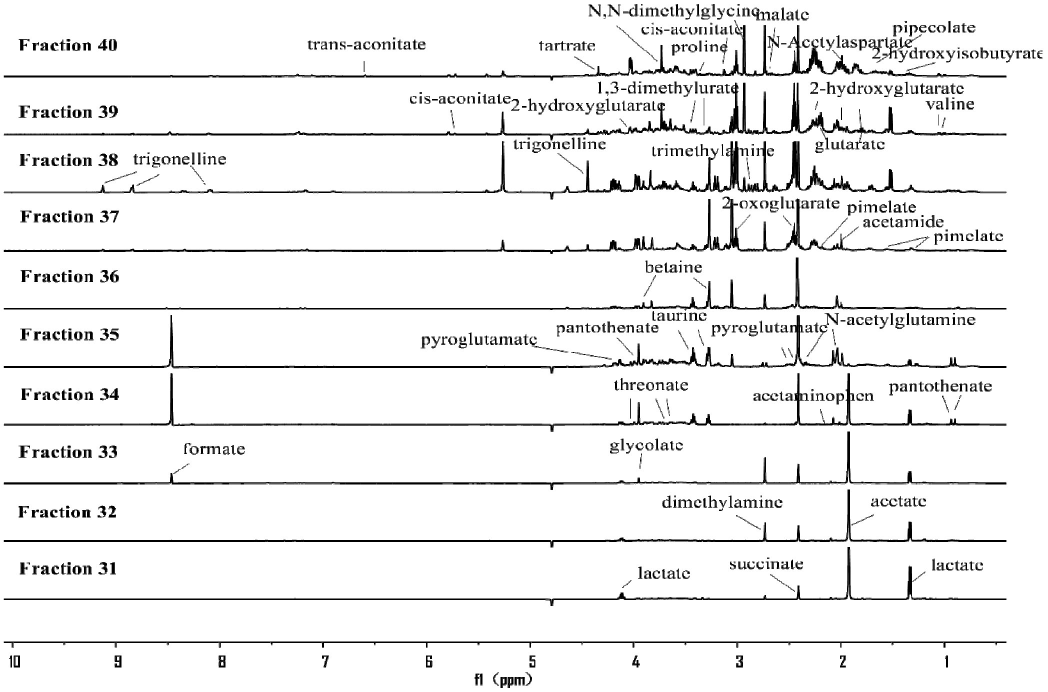

Fig. S4 NMR compositional analysis of rat urine HPLC fractions 31-40

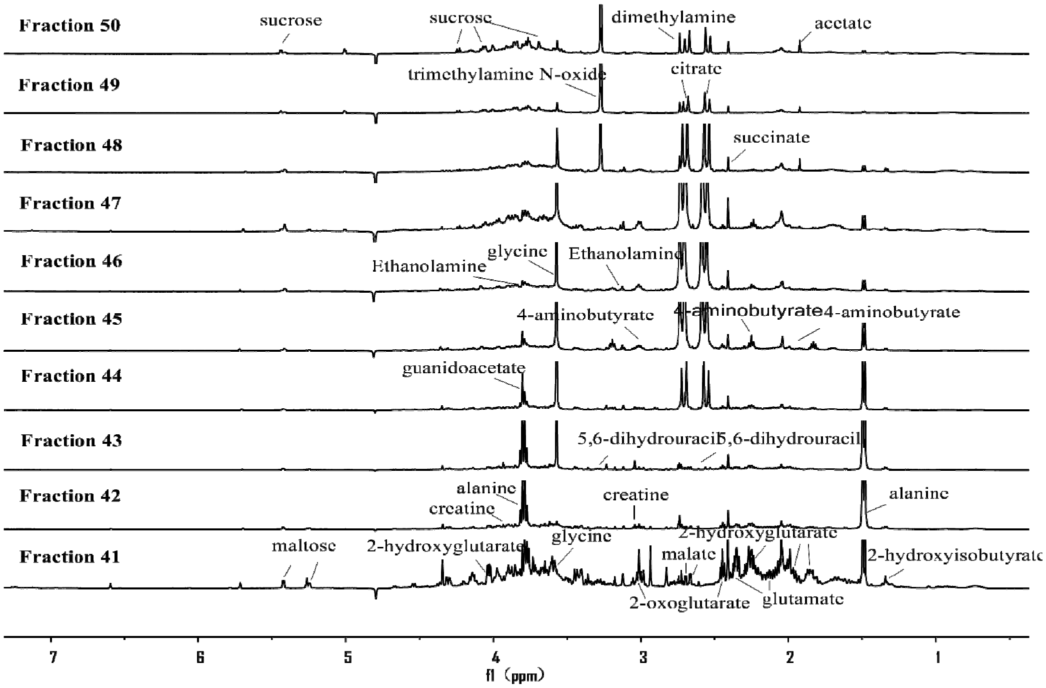

Fig. S5 NMR compositional analysis of rat urine HPLC fractions 41-50

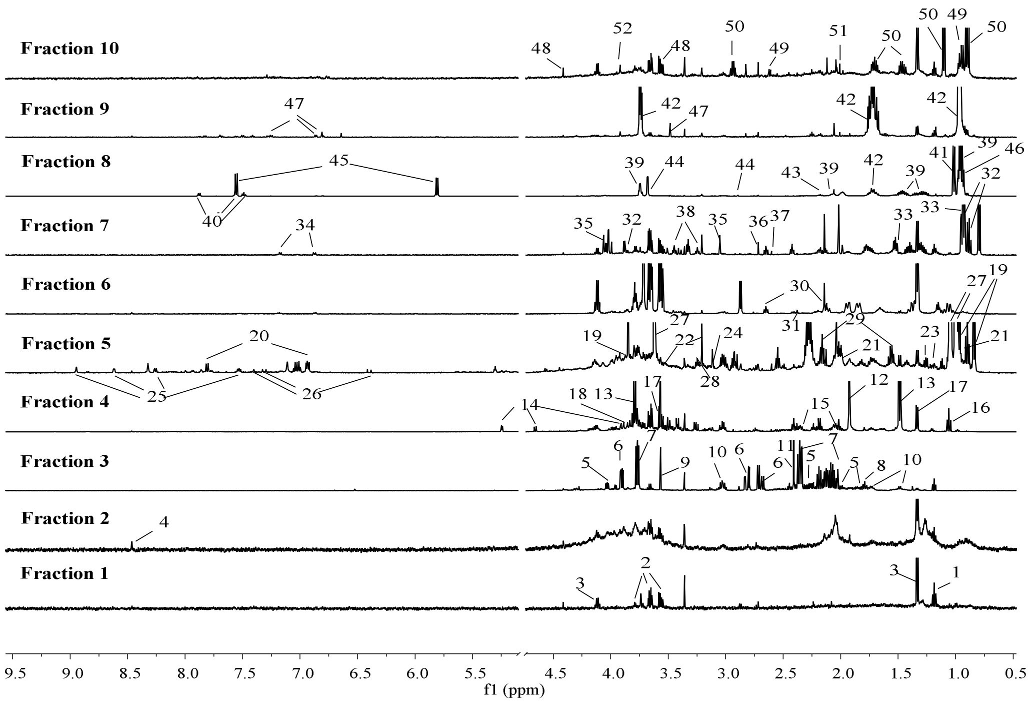

Fig. S6 NMR compositional analysis of rat feces HPLC fractions 1-10

1: ethanol, 2: glycerol, 3: lactate, 4: formate, 5: 2-hydroxyglutarate, 6: aspartate, 7: glutamate, 8: glutarate, 9: glycine, 10: lysine, 11: succinate, 12: acetate, 13: alanine, 14: glucose, 15: proline, 16: propionate, 17: Threonine, 18: Mannitol, 19: 2-hydroxyisovalerate, 20: 4-hydroxybenzoate, 21: butyrate, 22: choline, 23: fucose, 24: malonate, 25: nicotinate, 26: urocanate, 27: valine, 28: Arginine, 29: Pimelate, 30: methionine, 31: pyruvate, 32: 2-hydroxy-3-methylvalerate, 33: 2-hydroxyisocaproate, 34: 4-hydroxyphenylacetate, 35: creatinine, 36: dimethylamine, 37: methylamine, 38: taurine, 39: alloisoleucine, 40: benzoate, 41: isoleucine, 42: leucine, 43: suberate, 44: trimethylamine, 45: uracil, 46: Isovalerate, 47: 3-hydroxyphenylacetate, 48: 1,3-dihydroxyacetone, 49: 2-oxoisocaproate, 50: 3-methyl-2-oxovalerate, 51: acetamide, 52: glycolate

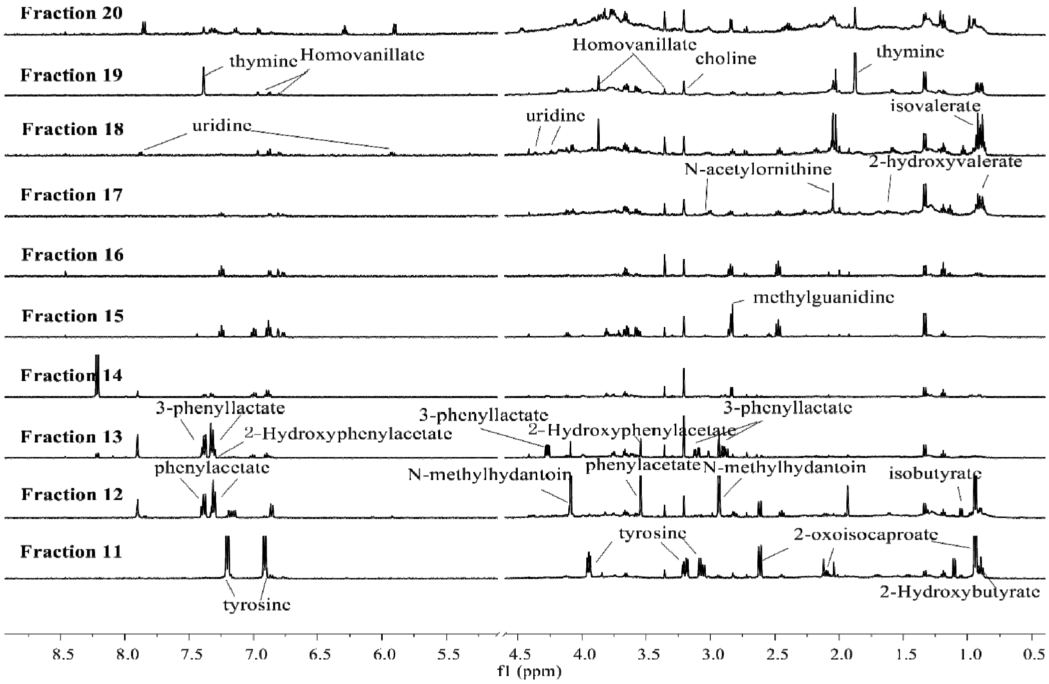

Fig. S7 NMR compositional analysis of rat feces HPLC fractions 11-20

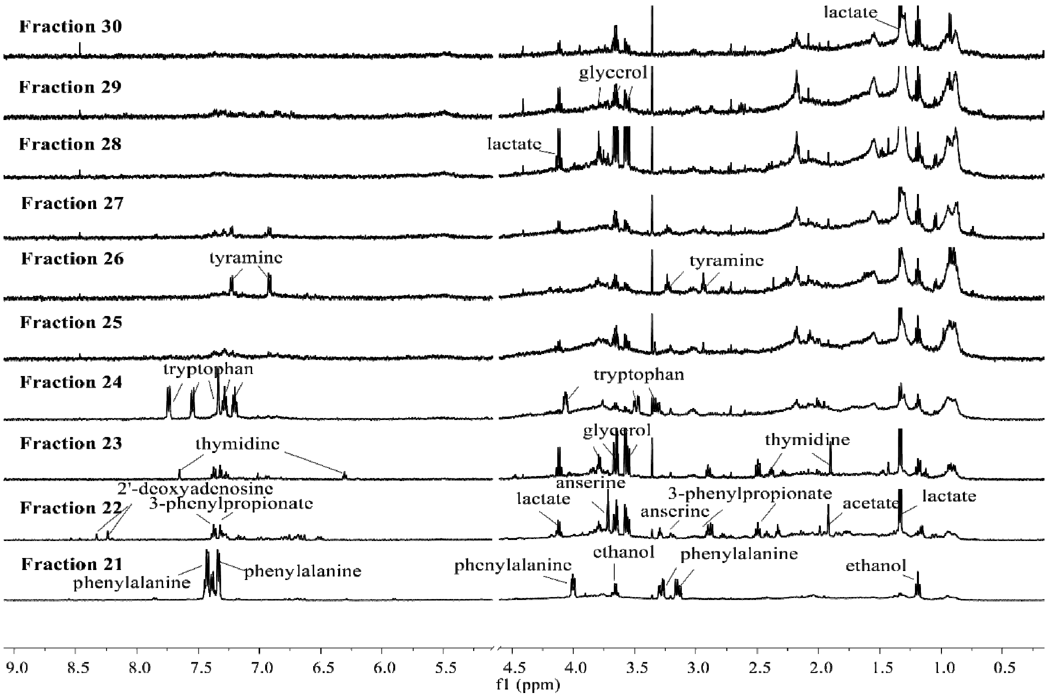

Fig. S8 NMR compositional analysis of rat feces HPLC fractions 21-30
